# Investigation on Polylactide (PLA)/Poly(butylene adipate-co-terephthalate) (PBAT)/Bark Flour of Plane Tree (PF) Eco-Composites

**DOI:** 10.3390/ma9050393

**Published:** 2016-05-19

**Authors:** Qiang Dou, Jun Cai

**Affiliations:** College of Materials Science and Engineering, Nanjing Tech University, Nanjing 210009, China; caijun0903@163.com

**Keywords:** poly(lactic acid), poly(butylene adipate-co-terephthalate), bark flour of plane tree, composite, morphology, mechanical property, crystallization

## Abstract

Polylactide (PLA)/poly(butylene adipate-co-terephthalate) (PBAT)/bark flour of plane tree (PF) eco-composites were prepared via melt blending. The morphologies, mechanical properties, crystal structures and melting and crystallization behaviors of the eco-composites were investigated by means of scanning electron microscopy (SEM), mechanical tests, polarized light microscopy (PLM), wide angle X-ray diffraction (WAXD) and differential scanning calorimetry (DSC), respectively. It is shown that the interfacial adhesion between PLA matrix and PF is weak and the mechanical properties of PLA/PF eco-composites are poor. The titanate treatment improves the adhesion between the matrix and the filler and enhances the stiffness of the eco-composites. The toughness is improved by PBAT and ductile fractured surfaces can be found. The spherulitic size of PLA is decreased by the addition of PF. The α crystalline form of PLA remains in the composites. Compared with PF, T-PF (PF treated by a titanate coupling agent) and PBAT have negative effects on the crystallization of PLA.

## 1. Introduction

In recent years, there are increasing concerns of global environmental problems and sustainable development. As one of the representative environmentally benign polymers, polylactide (PLA) is considered a replacement of some traditional petroleum-based polymers because it is produced from plant derived resources and has suitable properties [1,2]. It has been successfully used in packaging, biomedical, film and fiber applications. However, some inherent defects such as the low toughness, slow crystallization rate and relatively high cost have limited the application of PLA in some fields. Therefore, the modification of PLA has been investigated broadly. The toughness can be improved by plasticizers (e.g., tributyl citrate) [3,4] and polymers (e.g., poly(butylene adipate-co-terephthalate), PBAT) [3,5,6]. The crystallinity and crystallization rate can be accelerated by nucleating agents (e.g., dilithium hexahydrophthalate) [7,8,9]. The cost can be lowered by addition of fillers (e.g., starch) [4,6,10,11].

Nowadays, the demands for eco-composites from biodegradable polymers and natural fillers have attracted great interest in the industrial and academic research. Natural fillers are considered as inexhaustible raw materials for the increasing demand for environmentally friendly and biocompatible products. Natural fibers (the main component in most of them is cellulose) are renewable and cheaper substitutes of synthetic ones (glass, carbon and Kevlar fibers) and have several advantages, such as low cost, low density, reduced tool wear, acceptable specific strength properties, renewability and biodegradability. The primary occurrence of cellulose is the existing lignocellulosic material in forests, with wood as the most important source. Other cellulose-containing materials include agriculture residues, water plants, grasses, and other plant substances. Besides cellulose, they also contain hemicelluloses, lignin, and a comparably small amount of extractives. These green composites can be used in nondurable applications, in products with short life cycles, or in products used indoors, e.g., portable electronic device housings, packaging trays, food containers, and disposable utensils and cutlery. At the end of their service life, eco-composite products can be completely degraded in the environment or in composting units without harming the environment, or can be alternatively incinerated for energy recovery [12,13,14,15,16].

In the case of the eco-composites, various kinds of natural fibers such as kenaf fiber [17,18], sisal fiber [19], jute fiber [20], flax fiber [21,22], hemp fiber [23,24], phormium tenax fiber [25], pineapple leaf fiber [26], banana fiber [27], coconut fiber [28], durian skin fiber [29], cotton fiber [30], rice straw fiber [17,31], bamboo fiber [28,32], water bamboo husk [33], micro fibers separated from wheat husk and rye husk [34], fibers extracted from cuphea and lesquerella seeds [35], milkweed fiber [36], artichoke fiber [37], nutshells powders from almond, pistachio and walnut [38], micropowders derived from agricultural by-products such as oat husks, cocoa shells, and apple solids that remain after pressing [39], acorn powder [40], bleached birch kraft fiber [41], recycled newspaper cellulose fiber [42], fully bleached sulphite softwood pulp [43], and wood flours [28,44,45,46,47,48] are being added to biodegradable polymers, e.g., PLA to develop green composite materials. PLA biocomposites provide a means to produce relatively inexpensive PLA-based composites with a variety of properties.

The natural fiber/matrix interface plays an important role on the physical, chemical and mechanical properties of composites. To improve the interfacial properties, natural fibers are subjected to various physical and chemical treatments such as hydrothermal modification [47] sodium hydroxide (NaOH) treatment [19,23,24,26,27,29,45], NaOH + acetylation treatment [45], NaOH + silane treatment [45], silane treatment [18,19,24,26,27,40,47], aluminum acid ester coupling agent and stearic acid treatment [28], isocyanate treatment [32,40], butantetracarboxylic acid treatment [41], stearic acid treatment [47], maleic anhydride (MAH) treatment [47], MAH + dicumyl peroxide modification [17], enzyme modification [34], and with addition of chitosan [46], citrate esters [21], lignin [30], poly(butyl acrylate) (*in situ* suspension polymerization) [31], PLA grafted with MAH [40], and ethylene-methyl acrylate-glycidyl methacrylate (EMAGMA) [44]. The surface modification of natural fiber facilitates the fiber dispersion and induces bond formation between the fiber and the PLA matrix.

Platanus is a genus comprising a small number of tree species native to the Northern Hemisphere. Due to their decorative value, high growing rate, and beneficial effect on urban pollution, they mainly grow in parks or as sidewalk trees in Europe, East Asia, and North America. Three species, *i.e.*, *Platanus occidentalis*, *Platanus orientalis* and *Platanus* × *acerifolia* (a hybrid, plane tree) can be found in China. Millions of them are planted in China and the most widespread is the plane tree. The principal use of these trees is as ornamental trees, especially in urban areas and by roadsides. The mature barks peel off or exfoliate easily in irregularly shaped patches from the trunks spontaneously. Although some chemicals such as 3-acetylbetulinic aldehyde, betulonic acid, betulininic acid, daucosterol, 3,4,5-trimethoxyphenyl-*O*-β-d-glucopyranoside, and 3,4,5-trimethoxyphenyl-*O*-β-d-glucopyranoside-6’-sulfate sodium can be extracted from the barks and used as medicines [49,50]. Almost all of them are collected with other municipal wastes and are buried or incinerated. The research on bark flour of plane tree (PF) filled polymer composite has never been explored. In order to utilize this natural resource, the barks of plane tree are collected and processed as a novel natural filler for biodegradable polymers in our laboratory. They have potentials in furniture, automotives, household goods and packaging materials [51]. This article investigated the microstructures, mechanical properties, crystal structures and melting and crystallization behaviors of PLA/PBAT/PF eco-composites.

## 2. Results and Discussion

### 2.1. Characterization of PF

**Figure 1:**
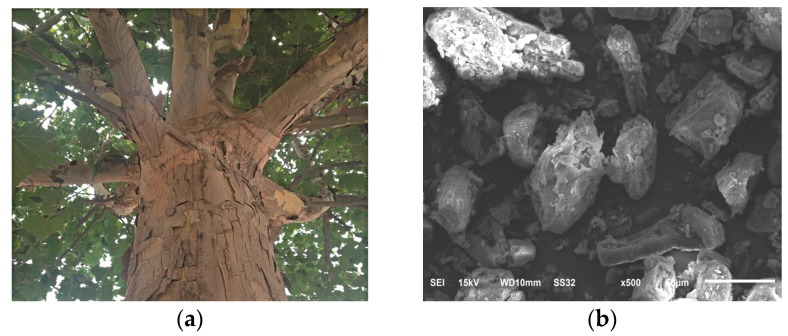
The photograph of the barks on the plane tree and the SEM micrograph of the bark flours. (**a**) barks; (**b**) bark flours.

The photograph of the barks on the plane tree and the SEM micrograph of the bark flours used in this study are shown in Figure 1. As can be seen, the bark flours have irregular and coarse surfaces. The particle size lies in a wide range, roughly from several to tens of microns.

**Figure 2:**
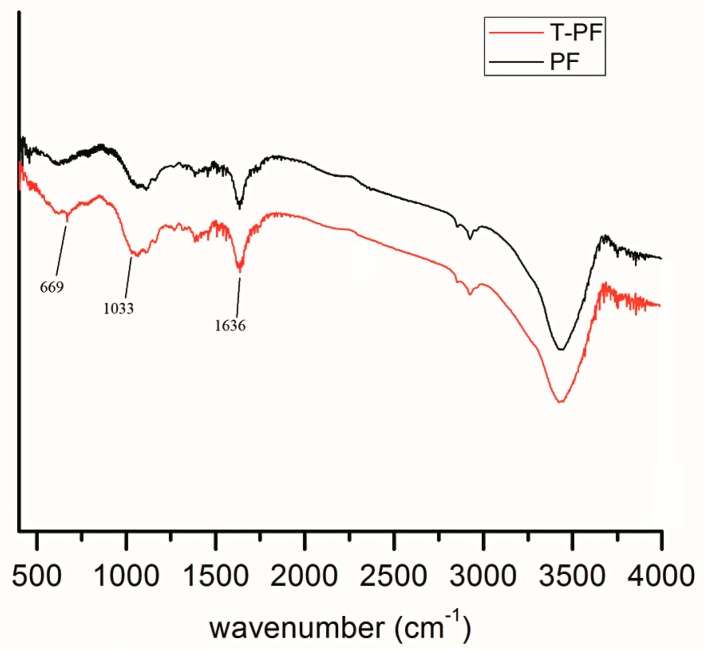
Fourier transform infrared spectra of PF and T-PF.

The Fourier transform infrared (FTIR) spectra of PF and T-PF are shown in Figure 2. Compared with PF, two weak new absorption bands (669 cm^−1^ for Ti–O and 1033 cm^−1^ for P–O) can be found for T-PF. The absorption bands between 1033 cm^−1^ and 1636 cm^−1^ are also strengthened. It may be referred that the titanate coupling agent (NDZ-311) reacted with the functional groups (e.g., hydroxyl) on the surface of PF. The hydrophilic surface of PF is changed and the compatibility between T-PF and PLA may be improved.

### 2.2. SEM Images of PLA Eco-Composites

**Figure 3:**
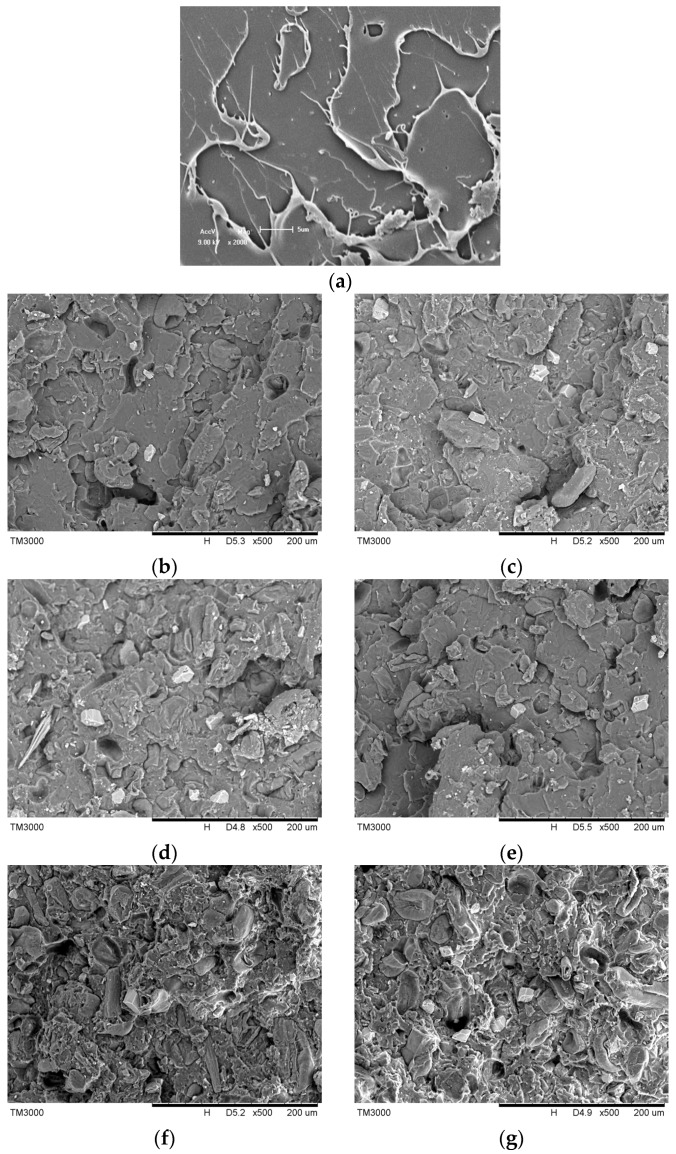
SEM micrographs of the fractured surfaces of the eco-composites. (**a**) neat PLA; (**b**) PLA/PF (90 wt %/10 wt %); (**c**) PLA/T-PF (90 wt %/10 wt %); (**d**) PLA/PF (70 wt %/30 wt %); (**e**) PLA/T-PF (70 wt %/30 wt %); (**f**) PLA/PBAT/T-PF (60 wt %/10 wt %/30 wt %); (**g**) PLA/PBAT/T-PF (50 wt %/20 wt %/30 wt %).

SEM micrographs of the fractured surfaces of the eco-composites are presented in Figure 3. The impact-fractured surface of neat PLA is smooth, which indicates that brittle fractures took place during the impact test. In PLA/PF composites, the fractured surface is very coarse, and detached PF particles can be found. In addition, some voids in the PLA matrix are visible where PF was located before the impact test. In addition, more voids can be found with the increase of PF contents. It reflects the poor adhesion between the hydrophobic PLA matrix and hydrophilic PF fillers.

**Figure 4:**
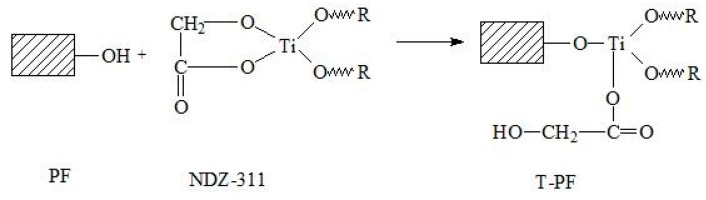
The coupling schematic of PF and titanate coupling agent (NDZ-311).

In the case of PLA/T-PF composites, the cavities of particle pull-out can also be found, but the amount decreases somewhat in comparison with that of PLA/PF composites, indicating that the titanate coupling agent promotes the interfacial adhesion between PLA and PF. The active groups of the coupling agent could react with the hydroxyl group of PF, weaken the hydrophilicity of PF and improve its compatibility with PLA matrix. Moreover, the long alkyl chain of the coupling agent could twist with the matrix. The coupling schematic is shown in Figure 4. Therefore, some “bridges” were formed between PLA and PF in the composites, which improve the interfacial interaction. Mamum and Bledzki [34] found that the enzyme modification improved the fiber-matrix adhesion in wheat and rye husks/PLA composites. Wang *et al.* [44] also found weak interface interaction in PLA/wood flour biocomposites. The silane coupling agent and EMAGMA could improve the adhesion between PLA and wood flours.

For PLA/PBAT/T-PF composites (Figure 3f,g), detached PF particles and cavities can still be found. However, the yield deformations (stress whitening regions) appear with the increase of PBAT content, indicating that plastic deformation occurred and are related to the addition of PBAT.

### 2.3. Mechanical Properties of PLA Eco-Composites

**Figure 5:**
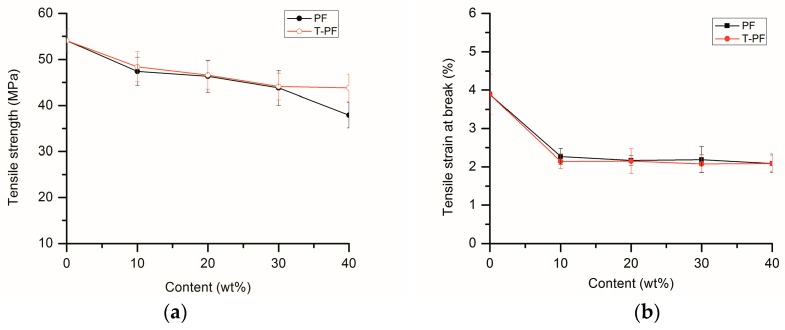

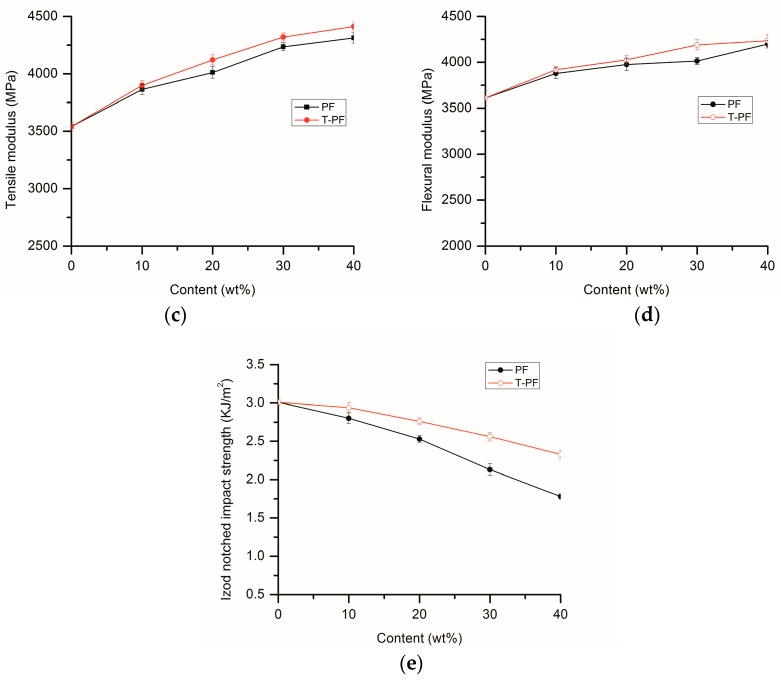
Mechanical properties of the eco-composites. (**a**) tensile strength; (**b**) tensile strain at break; (**c**) tensile modulus; (**d**) flexural modulus; (**e**) Izod notched impact strength.

Figure 5 presents the mechanical properties of PLA eco-composites. For PLA/PF and PLA/T-PF composites, the tensile strength, tensile stain at break and Izod notched impact strength decrease with the increasing content of the filler. This may imply that there is a poor interfacial interaction between the filler and the matrix because of the poor stress transformation across interphase. The poor interfacial adhesion is proven by the SEM images (Figure 3).

It was found that the addition of wood flour to the PLA matrix significantly increased the tensile modulus and flexural modulus because the modulus of wood fiber is higher than that of the polymer [46]. The tensile modulus and flexural modulus of the eco-composites continuously increases to the maximum at the loading of 40% filler in this study. The increases of tensile (Young’s) modulus were also found in PLA/banana fiber biocomposites [27], cotton fiber-reinforced PLA composites [30], PLA/bamboo fiber biocomposites [32], and PLA/artichoke fiber composites [37]. This phenomenon is common in composites reinforced with hard fillers.

PLA/T-PF composites show appreciably higher tensile modulus, flexural modulus and Izod notched impact strength than those of PLA/PF composites. It is evident that the titanate coupling agent improves the interfacial bonding between PF and PLA and increases the mechanical properties. Wang *et al.* [44] also found that the silane coupling agent had the positive influence on the mechanical properties of PLA/wood flour biocomposites.

For PLA/PBAT/T-PF composites (Table 1), the tensile strength, tensile modulus and flexural modulus decrease with the increasing content of PBAT. However, the tensile stain at break and Izod notched impact strength increase continuously with PBAT content. It means that the elastomeric PBAT improves the toughness but weakens the strength and stiffness of the composites. The ductile fractured surfaces can be found in Figure 3f,g.

### 2.4. Polarized Light Microscopy Images of PLA Eco-Composites

**Figure 6:**
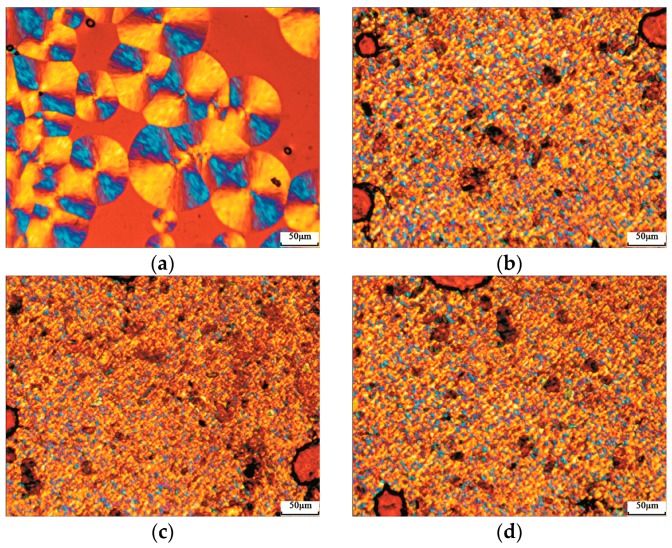
Polarized light images of isothermally crystallized samples. (**a**) PLA; (**b**) PLA/PF (70 wt %/30 wt %); (**c**) PLA/T-PF (70 wt %/30 wt %); (**d**) PLA/PBAT/T-PF (60 wt %/10 wt %/30 wt %).

Figure 6 shows polarized light microscopy (PLM) images of the isothermally crystallized samples. The spherulitic morphology of the neat PLA sample presents the perfect Maltase extinction crosses, and the spherulitic size is larger than 100 μm. Crystallization induced by self nucleation is possible, and the spherulites grow outwards until they impinge on each other and further growth is arrested.

In comparison to the neat PLA sample, PF and their aggregates can be observed in the eco-composites. As a result of the nucleating effect of PF, the spherulitic size of PLA decreases and the number of spherulites increases greatly. Because of the disturbance of the filler, the fine textures of the spherulites of PLA are difficult to be distinguished in the composites. The similar results were also found in PLA/CaCO_3_ [4] and PLA/nucleating agents systems [7,8,9].

### 2.5. Wide Angle X-ray Diffraction Characterization of PLA Eco-Composites

**Figure 7:**
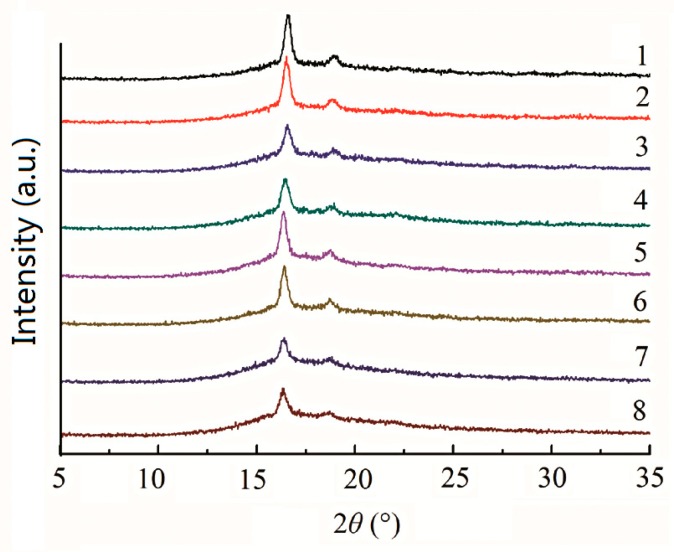
Wide angle X-ray diffraction patterns of isothermally crystallized samples. (**1**) PLA; (**2**) PLA/PF (90 wt %/10 wt %); (**3**) PLA/T-PF (90 wt %/10 wt %); (**4**) PLA/PF (80 wt %/20 wt %); (**5**) PLA/T-PF (80 wt %/20 wt %); (**6**) PLA/PF (70 wt %/30 wt %); (**7**) PLA/T-PF (70 wt %/30 wt %); (**8**) PLA/PBAT/T-PF (60 wt %/10 wt %/30 wt %).

Figure 7 illustrates wide angle X-ray diffraction (WAXD) patterns of the isothermally crystallized samples. The diffraction peaks at 2θ = 16.7° and 19.3° correspond to (110)/(200) and (203) planes of PLA, respectively, which belong to the orthorhombic α crystal form of PLA [4,17,23,33,39]. Only two distinctive peaks of α-PLA are observed for PLA/PF, PLA/T-PF and PLA/PBAT/T-PF composites. No obvious differences related to the diffraction peaks, and peak positions are observed between the neat PLA and its composites. Therefore, the crystal structure of PLA is not changed in the eco-composites.

### 2.6. DSC Measurements of PLA Eco-Composites

**Figure 8:**
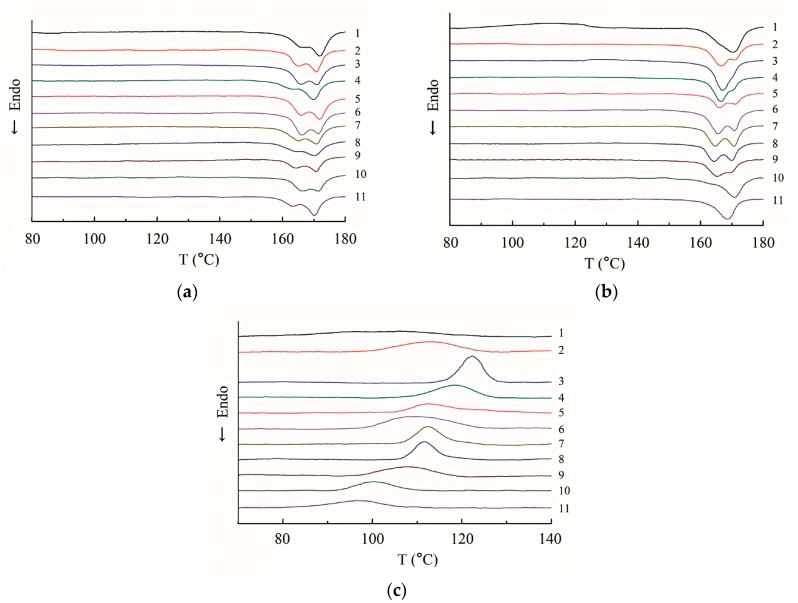
Differential scanning calorimetry curves of isothermally crystallized samples. (**1**) PLA; (**2**) PLA/PF (90 wt %/10 wt %); (**3**) PLA/PF (80 wt %/20 wt %); (**4**) PLA/PF (70 wt %/30 wt %); (**5**) PLA/PF (60 wt %/40 wt %); (**6**) PLA/T-PF (90 wt %/10 wt %); (**7**) PLA/T-PF (80 wt %/20 wt %); (**8**) PLA/T-PF (70 wt %/30 wt %); (**9**) PLA/T-PF (60 wt %/40 wt %); (**10**) PLA/PBAT/T-PF (60 wt %/10 wt %/30 wt %); (**11**) PLA/PBAT/T-PF (50 wt %/20 wt %/30 wt %).

Figure 8 shows the heating and cooling curves of the isothermally crystallized samples. The differential scanning calorimetry (DSC) parameters are listed in Table 2. The first heating curves are shown in Figure 8a, and double melting peaks are found for neat PLA and PLA eco-composites. Many semi-crystalline polymers show multiple melting peaks due to bimodal crystal distributions produced by annealing, reorganization and recrystallization, polymorphism and different molecular weight species [52]. This phenomenon is also found for neat PLA and PLA blends and composites in the literatures [3,7,8,9,18,23,25,27,31,33,38,39]. The WAXD spectra (Figure 7) indicate that only one crystalline modification was developed in the samples and the double melting peak seen in the DSC heating run may reflect the melting of crystalline regions of various sizes and perfection. The low- and high-temperature peaks are ascribed to the melting of the crystals with two distinct lamellar populations. In addition, the high-temperature peaks may also originate from the melting of crystals formed through a melt-recrystallization process during the heating scan. *X*_c_ values of PLA/PF and PLA/T-PF composites are greater than that of neat PLA because PF and T-PF may act as nucleating agents and increase the crystallinity of PLA. Moreover, *X*_c_ of PLA/PF composites is slightly higher than that of PLA/T-PF composites at each filler loading. It is considered that the molecular motion of the polymer matrix could be restricted by the addition of coupling agent. In our system, the functional group of titanate coupling agent and PLA molecules may form “bridges” and the molecular motion of PLA could be restricted, results in a decrease of *X*_c_ in PLA/T-PF composites. Lee and Wang [32] also found that the enthalpy of crystallization decreased by increasing coupling agent content in PLA/bamboo fiber composites. *X*_c_ values of PLA/PF and PLA/T-PF composites both reach the maxima at 20 wt % filler contents, then they decrease with the increasing contents of fillers, and the values of the former is greater than those of the latter. Maybe excess fillers restrict the molecular motion of the matrix, and then hamper the crystallization ability of the polymer. *X*_c_ values of PLA/PBAT/T-PF samples decrease with the increasing PBAT content. As a flexible polymer, PBAT may decrease the crystallinity of PLA and weakens the nucleating effect of T-PF.

Figure 8b shows the second heating curves of the isothermally crystallized samples. A wide cold crystallization peak (80–130 °C) appears for neat PLA, indicating the incomplete crystallization of PLA (*X*_c_ = 15.24%) during the cooling process. Compared with the first melting curve, the low temperature peak almost disappears and the high temperature peak becomes bigger for neat PLA. Obviously, the crystals formed during the cold crystallization melted later than those formed after cooling process. In addition, the low temperature peaks become smaller or disappears for PLA/PBAT/T-PF samples. Maybe the perfect crystals are the major ones after the cooling procedures. Whereas the opposite trends show for PLA/PF and PLA/T-PF composites, the low temperature peaks strengthen during the second heating procedure, indicating that the impeferct crystals predominate after the cooling procedure. It may relate to the nucleating effect of the fillers, the imperfect crystals were facilitated to form on the surfaces of the fillers, and the perfect ones were suppressed during the cooling procedure. The trends of *X_c_* values of the samples are similar to those in the first melting procedure, as Table 2 shows. However, these values are lower than those in the first melting procedure. It owes to the isothermal crystallized samples used in the first melting procedure, thus the increased crystallinities are present.

Figure 8c shows the cooling curves of the isothermally crystallized samples. It is difficult to identify the wide crystallization peak of neat PLA. It reflects the slow crystallization rate and poor crystallization ability of neat PLA. Obvious crystallization peaks appear for PLA/PF and PLA/T-PF samples, and *T*_c_ values reach the maxima at 20 wt % fillers. *T*_c_ values of PLA/PF composites are greater than those of PLA/T-PF samples. These results are in accordance with *X_c_* values in Table 2. Excess fillers may restrain the mobility of PLA chains and disturb the crystallization of PLA. *T*_c_ values of PLA/PBAT/T-PF composites are less than that of a PLA/T-PF (70/30) sample, and *T*_c_ decreases with the increasing PBAT contents. It indicates that PBAT plays a negative effect on the crystallization of PLA.

## 3. Materials and Methods

### 3.1. Materials

**Figure 9:**
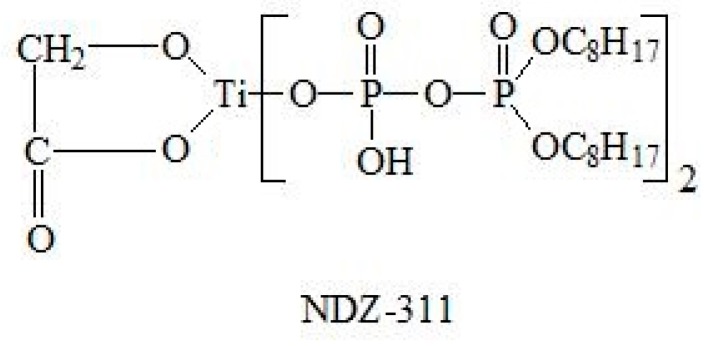
The structural formula of NDZ-311.

The commercial PLA pellets (Ingeo™ 4032D, Nature Works, Minnetonka, MN, USA) exhibits a density of 1.25 g/cm^3^, viscosity-average molecular weight of 13.6 × 10^4^ [8]. Titanate coupling agent (NDZ-311, bis(P,P-bis-ethylhexyl diphosphato) ethanediolato titanate, CAS no. 65467-75-6, the structural formula is shown in Figure 9) was obtained from Nanjing Shuguang Chemical Co., Ltd. (Nanjing, China). Petroleum ether (Wuxi Yasheng Chemical Co., Ltd., Wuxi, China) was commercially available. All were used as received.

### 3.2. Preparation of Samples

Barks of plane trees were collected in the campus of Nanjing Tech University. They were washed with tap water and dried in a blast oven at 100 °C for 4 h. Then, they were smashed into flours in a high-speed pulverizer and passed through a 100 mesh (150 μm) sieve. The images of the barks and PF are shown in Figure 1. In addition, 100 g of PF and a solution of 1 g titanate coupling agent in 20 g petroleum ether were added into a high-speed blender. The mixture was stirred at 25,000 rpm for 2 min. Then, the mixture was dried to a constant weight at 100 °C. The treated PF was labeled as T-PF.

Before use, PLA, PF and T-PF were dried under vacuum at 80 °C for 8 h. PLA/PF (T-PF) composites with weight content ratios 90/10, 80/20, 70/30, 60/40 and PLA/PBAT/T-PF composites with weight content ratios 60/10/30 and 50/20/30 were prepared. The mixtures were compounded in an internal mixer (HL200, Scientific Instruments Factory of Jilin University, Changchun, China) at 185 °C and 40 rpm for 5 min, then they were injection molded into test specimens in a micro-ram injection molding machine (SZ-15, Wuhan Rui Ming Plastic Machinery Company, Wuhan, China) at 200 °C and 0.8 MPa.

A granule of the eco-composites was placed between two glass slides on a hot stage kept at 200 °C for 5 min to allow the sample to melt completely and remove thermal history, and then squeezed on the top slide to form a film and quickly transferred onto a hot stage kept at 120 °C and lasted for 60 min. Finally, the film was quenched to room temperature. The thickness of the samples after isothermal crystallization was about 0.5 mm for DSC and WAXD characterization, and 10–20 μm for PLM observation, respectively.

### 3.3. Scanning Electron Microscopy (SEM) Observations

The PF particles and impact-fractured surfaces of the selected specimens were sputtered with gold under vacuum. Then, the surfaces were observed using SEM (TM-3000, Hitachi, Tokyo, Japan).

### 3.4. Fourier Transform Infrared Spectroscopy (FTIR) Characterizations

The FTIR spectra of PF and T-PF were recorded with a FTIR spectrometer (Nexus 670, Thermo-Nicolet, Madison, WI, USA) using KBr pellets over the range from 400 to 4000 cm^−1^.

### 3.5. Mechanical Measurements

The tensile strength and flexural modulus of the specimens were tested using an electromechanical universal testing machine (CMT5254, Shenzhen SANSI Testing Machine Co. Ltd., Shenzhen, China) according to ISO 527 and ISO178, respectively. The Izod notched impact strength was tested using a pendulum impact testing machine (MZ2056, Jiangdu Mingzhu Testing Machine Factory, Jiangdu, China) according to ISO180. Before impact measurement, specimens were notched on a plastic specimen notcher (MZ2061, Jiangdu Mingzhu Testing Machine Factory, Jiangdu, China). All specimens were conditioned at 23 °C for 24 h before tests.

### 3.6. Polarized Light Microscopy (PLM) Observations

Spherulitic morphologies of the isothermally crystallized samples were observed using a polarized light microscope (LW-200-4JS, Shanghai LW Scientific Co., Ltd., Shanghai, China) equipped with cross polars and a CCD camera. A primary red filter was located diagonally between cross polars. Pictures were captured and stored in a computer.

### 3.7. Wide Angle X-ray Diffraction (WAXD) Characterization

WAXD diffraction patterns of the isothermally crystallized samples were recorded using an X-ray diffractometer (ARL X’TRA, Thermo Electron Corp., Milford, MA, USA) using Cu K_α_ radiation (λ = 0.154 nm). It was operated in reflection mode at 40 kV and 30 mA. Radial scans of intensity *versus* diffraction angle (2θ) were recorded in the range of 5° to 35° with a scanning rate of 10°/min and a step size of 0.02°.

### 3.8. Differential Scanning Calorimetry (DSC) Measurements

The melting and crystallization behaviors of the isothermally crystallized samples were measured with a ZF-DSC-D2 DSC apparatus (Shanghai Zufa Industry Co., Ltd., Shanghai, China) in a dry nitrogen atmosphere. The instrument was calibrated with pure indium, tin and zinc for temperature and heat flow, respectively. All samples were heated from room temperature to 200 °C at a rate of 10 °C/min, and held at 200 °C for 5 min to eliminate the thermal history. Then, the melted samples were cooled down to 30 °C at a rate of 5 °C/min and held at 30 °C for 5 min, finally reheated to 200 °C at the rate of 10 °C/min. The melting and cooling curves were recorded. The melting temperature (*T*_m_), enthalpy of melting (∆*H*_m_) and enthalpy of cold crystallization (∆*H*_cc_) were determined from the melting curves. The peak temperatures (*T*_c_) of melt-crystallization were determined from the cooling curves.

The crystallinity (*X_c_*) of the samples was calculated using Equation (1):

*X_c_* = 100% × (∆*H*_m_ − ∆*H*_cc_)/Δ*H*^°^_m_ W_PLA_, (1)

where Δ*H*^°^_m_ = 93.6 J/g for 100% crystalline PLA, W_PLA_ is the wt % of PLA in the composite [4].

## 4. Conclusions

The morphology, mechanical properties and crystallization of PLA/PF, PLA/T-PF and PLA/PBAT/T-PF eco-composites were investigated. SEM results present a poor interfacial adhesion between PLA matrix and PF, while titanate treatment improves the interfacial adhesion. The ductile fracture mode is found in PLA/PBAT/T-PF composites. The tensile modulus and flexural modulus are improved by the fillers, while tensile strength, tensile stain at break and Izod notched impact strength show an opposite trend. Titanate coupling agent improves the mechanical properties of PLA/PF composites. PBAT improves the toughness but weakens the strength and stiffness of the eco-composites. PLM observations show that PF and T-PF significantly decrease the size of spherulites. WAXD results show that the crystal structure of PLA is not changed in the composites. DSC results indicate that PF remarkably improves the crystallization of PLA, but T-PF and PBAT weaken the crystallization ability of the composites.

## Figures and Tables

**Table 1:** Mechanical properties of PLA/PBAT/T-PF composites.

PLA/PBAT/T-PF	Tensile Strength (MPa)	Tensile Strain at Break (%)	Tensile Modulus (MPa)	Flexural Modulus (MPa)	Izod Notched Impact Strength (KJ/m^2^)
70/0/30	44.1 ± 2.9	2.08 ± 0.23	4320 ± 35	4189 ± 56	2.56 ± 0.16
60/10/30	43.4 ± 3.8	2.76 ± 0.35	4221 ± 43	4089 ± 32	3.11 ± 0.24
50/20/30	40.2 ± 3.1	3.54 ± 0.44	4108 ± 37	3989 ± 39	3.82 ± 0.33

**Table 2:** Differential scanning calorimetry parameters of isothermally crystallized samples.

Samples	First Melting		Second Melting		Cooling
	*T*_m_ (°C)	*X_c_* (%)	*T*_m_ (°C)	*X_c_* (%)	*T*_c_ (°C)
Neat PLA	165.9, 171.7	20.87	165.3, 170.2	15.24	-
PLA/PF (90/10)	165.0, 170.8	41.67	166.6, 170.8	40.19	112.7
PLA/PF (80/20)	165.8, 171.0	49.86	166.9, 170.5	47.84	122.3
PLA/PF (70/30)	163.0, 170.0	45.36	166.5, 170.2	44.23	118.5
PLA/PF (60/40)	165.9, 171.9	44.28	165.9, 171.2	43.86	112.3
PLA/T-PF (90/10)	166.2, 171.6	40.17	165.5, 170.8	40.01	109.2
PLA/T-PF (80/20)	165.1, 170.9	47.28	164.5, 170.5	45.36	112.3
PLA/T-PF (70/30)	164.3, 170.0	43.76	164.2, 170.0	42.94	111.5
PLA/T-PF (60/40)	164.2, 170.7	42.15	165.2, 169.7	40.73	107.5
PLA/PBAT/T-PF (60/10/30)	166.1, 171.5	35.69	163.3, 170.8	32.15	100.4
PLA/PBAT/T-PF (50/20/30)	163.1, 170.1	30.75	168.6	28.85	96.6
